# All-Dielectric Transreflective Angle-Insensitive Near-Infrared (NIR) Filter

**DOI:** 10.3390/nano12152537

**Published:** 2022-07-23

**Authors:** Ayesha Shaukat, Rahila Umer, Frazer Noble, Khalid Mahmood Arif

**Affiliations:** 1 Department of Mechanical and Electrical Engineering, SF&AT, Massey University, Auckland 0632, New Zealand; a.shaukat@massey.ac.nz (A.S.); f.k.noble@massey.ac.nz (F.N.); 2 Engineering and Management Sciences, Balochistan University of Information Technology, Quetta 87100, Pakistan; rahilaumer@buitms.edu.pk

**Keywords:** NIR filter, thin films, angle invariance, FDTD, spectroscopic ellipsometry

## Abstract

This paper presents an all-dielectric, cascaded, multilayered, thin-film filter, allowing near-infrared filtration for spectral imaging applications. The proposed design is comprised of only eight layers of amorphous silicon (A-Si) and silicon nitride (Si${}_{3}$N${}_{4}$), successively deposited on a glass substrate. The finite difference time domain (FDTD) simulation results demonstrate a distinct peak in the near-infrared (NIR) region with transmission efficiency up to 70% and a full-width-at-half-maximum (FWHM) of 77 nm. The theoretical results are angle-insensitive up to $60^{{}^{\circ}}$ and show polarization insensitivity in the transverse magnetic (TM) and transverse electric (TE) modes. The theoretical response, obtained with the help of spectroscopic ellipsometry (SE), is in good agreement with the experimental result. Likewise, the experimental results for polarization insensitivity and angle invariance of the thin films are in unison with the theoretical results, having an angle invariance up to $50^{{}^{\circ}}$.

## 1. Introduction

The unique spectral signature of molecular objects allows object differentiation and detection via noncontact spectral imaging devices. Unlike the human eye, which is limited to the visible regime of the spectrum, spectral imaging helps collect image data within specified wavelength ranges in the electromagnetic spectrum. The interest of researchers has been lately diverted to the infrared (IR) region due to its high demand in thermal imaging sensors [1,2,3,4], medical endoscopes [5,6], agricultural aerial view imaging cameras [7,8], and security and surveillance [9,10] cameras, etc. In near-infrared (NIR) filtering, a narrow band is highly desired for improving the spatial resolution in hyperspectral imaging. Likewise, the cost and ease of fabrication also play an imperative role in choosing the sensing device. Moreover, its compatibility with the existing complementary metal-oxide semiconductor (CMOS) imaging device is also essential.

Subwavelength gratings have received great attention due to their small footprint, narrow bandwidth, spectral tunability, and vast applications [11,12]. There have been numerous NIR-based designs reported which include guided-mode resonance (GMR)-based resonators [13], Fabry–Pérot interferometers [14], and plasmonic absorbers [15,16].

A mid-infrared (IR) spectral filter based on GMR was studied in [17]. It contained etched silicon nitride (Si${}_{3}$N${}_{4}$) gratings on a Si${}_{3}$N${}_{4}$ layer, which, in this particular case, acted as a waveguide layer with a soda lime substrate at one of its ends. The introduced periodic modulation allowed phase matching of an external incident beam into modes that re-radiated to “leaky modes”. This resulted in reflectance spectra with full-width-at-half-maximum (FWHM) measurements as low as 33 nm, with a tunable peak in the mid-IR region. Nevertheless, the design was highly sensitive to incident and polarization angles of incident light.

Shah et al. [18] demonstrated that the subwavelength circular and elliptical nanohole array milled onto a gold (Au) film exhibits high transmission efficiency, polarization insensitivity, and narrow bandwidth, which is highly desirable for narrow pass-bands, e.g., those found in multispectral imaging. The obtained narrow bandwidth is accredited to the Fano resonance [19]. Their experimental study showed transmission efficiency up to 44%, and the FWHM was 79 nm. The fabrication of circular and elliptical holes was performed through electron beam lithography (EBL) [20]. However, the design was not CMOS-compatible.

A very strong magnetic resonance in metamaterials was theoretically demonstrated for a highly sensitive refractive index sensor in [21]. It comprised a periodic array of closely spaced silver (Ag)-based nanodisks placed on a Ag substrate. The strong magnetic resonance is an antisymmetric resonant mode that arises from the near-field plasmon hybridization within the pair of metal nanodisks. The studied metamaterials had very high sensitivity and figure of merit (FOM), which made them potential candidates for label-free sensing. However the narrow bandwidth paved the way for other applications.

Recently, a theoretical and experimental study of a square array of ultra-thin A-Si nanopillars demonstrated polarization-independent, narrowband, NIR spectral filtering via GMR [22]. The nanopillars were embedded in silicon dioxide (SiO${}_{2}$) and the fabrication process involved deposition of A-Si on the substrate using plasma-enhanced chemical vapor deposition (PECVD) [23]. The nanopillars were obtained through EBL and were later in-filled with a SiO${}_{2}$ layer. The results attained ≈90% reflectivity with a 20 nm FWHM. Similarly, the spatial resolution of multispectral filters was addressed in a one-dimensional A-Si GMR array [24]. It has been observed that the higher the number of gratings in a diffraction grating, the lower the FWHM will be [25]; however, in this particular case, thin aluminum (Al) films were used as mirrors, which allowed the finite array to approximate an infinite array and enabled small footprint for NIR applications.Although the mentioned examples produced the desired results, the fabrication process required multiple steps and increased the overall design cost.

On the contrary, thin film design have received the attention of researchers because, in general, the fabrication steps in thin films are minimal and the fabricated/deposited films are insensitive to light polarization. However, the films show high dependency on the incident angle of light. This issue has been addressed by many researchers [26,27,28,29] using highly refractive index materials, such as A-Si, that allows high angle invariance by blocking a big portion of the visible region. In another approach, Ji et al. [30] used a seven-layer stack of Si${}_{3}$N${}_{4}$ and A-Si, which blocked the visible region but allowed NIR transmission. In the visible region, the blocking of the light is a drawback; however, this could be a useful feature for NIR filtering. Therefore, here, we manipulated the thicknesses and the number of layers in the stack to demonstrate NIR filtering with high transmission efficiency and narrow bandwidth. A summary of the discussed examples and other reported works is provided in Table 1.

## 2. Design Methodology

**Figure 1:**
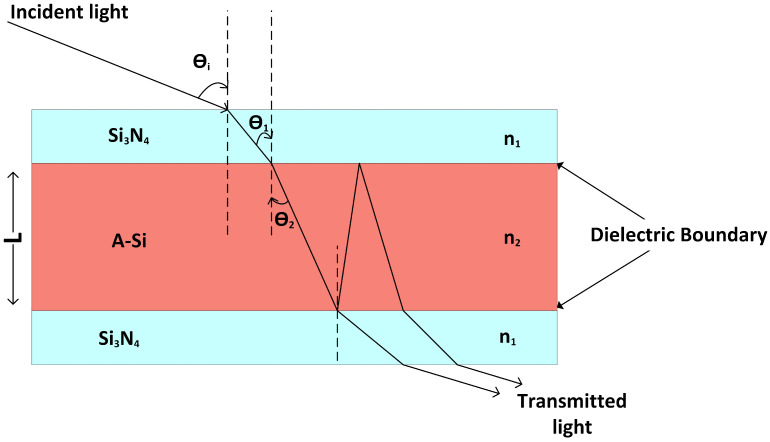
Response of incident light in 1D ternary photonic crystal with low (Si${}_{3}$N${}_{4}$) and high A-Si refractive indices layers.

In this section, a ternary cavity is studied to understand the behavior of incident light impinged at an angle $\theta_{i}$ in A-Si cavity sandwiched between Si${}_{3}$N${}_{4}$ layers, as depicted in Figure 1.

According to Fresnel’s equation, the periodic peak transmission (*T*) of the refracted light through the cavity is given as

(1) $$T=\frac{{\left(1-R\right)}^{2}}{{\left(1-R\right)}^{2}+4R\sin^{2}\left(\frac{n_{2}L\cos\theta_{2}\pi }{\lambda +2\phi }\right)},$$

where the angle of refraction ($\theta_{1}$) due to the Si${}_{3}$N${}_{4}$–air interface is determined with the help of Snell’s law. *R* is the induced reflectance comes from the partially reflecting the Si${}_{3}$N${}_{4}$–A-Si interface; $\theta_{2}$ is the propagation angle inside the A-Si cavity; λ is the wavelength; $n_{2}$ is the refractive index of A-Si cavity; ϕ the phase shift due to internal reflection at the interface [35]. The optical thickness of the cavity at different angles can be expressed as follows

(2) $$\delta =\frac{2\pi n_{2}L\cos\theta_{i}}{m\lambda },$$

where *m* is the number of resonating modes inside the cavity. It can be implied from Equation (2) that δ reduces with the increase in angle of incident light. The resonant wavelength of cavity is given as follows

(3) $$\lambda_{o}=2\pi L\sqrt{n_{2}^{2}-\cos\theta_{i}^{2}}.$$

The change in phase shift at the interface causes fractional wavelength shift ($\frac{\delta \lambda_{o}}{\lambda_{o}}$), and the rate of change in fractional wavelength shift with respect to $\theta_{i}$ is inversely proportional to the refractive index of the cavity. Mathematically, it is represented as

(4) $$\frac{\partial (\frac{\delta \lambda_{o}}{\lambda_{o}})}{\partial \theta_{i}}=\frac{-2L\sin\theta_{i}\cos\theta_{i}}{\sqrt{n_{2}^{2}-\sin\theta_{i}^{2}}}.$$

It can be deduced from Equation (4) that high-refractive-index materials show negligible wavelength shift with respect to the change in the incident angle. Similarly, there have been many reported examples where materials with a high refractive index [27,36] are used to overcome the angle variance of output signals. Hence, in the light of the above evidences, it can be deduced that A-Si is the right choice for the proposed design [37,38].

**Figure 2:**
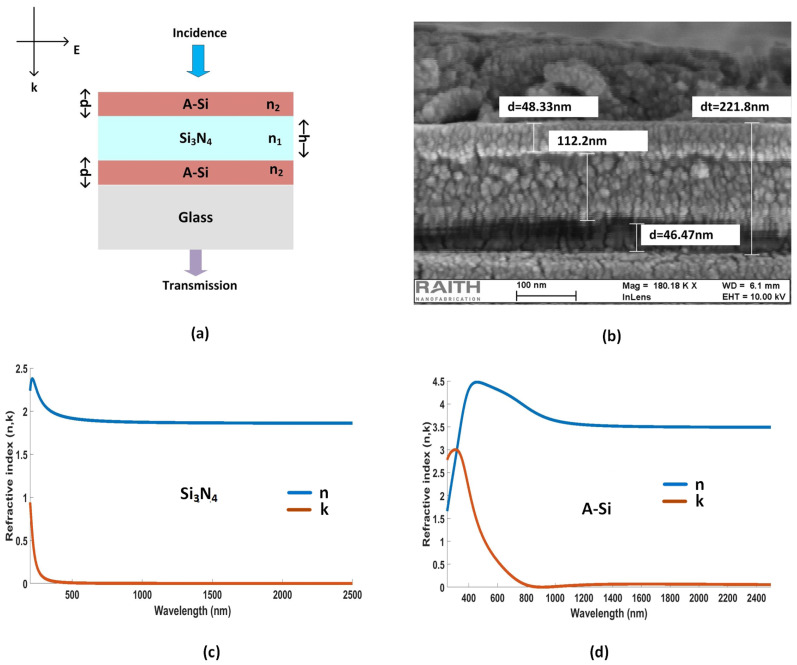
(**a**) A hybrid etalon when cavity thickness $d_{u}=d_{l}$ = *d* = 40 nm and h=100 nm. (**b**) SEM image of hybrid etalon. (**c**) Refractive indices of silicon nitride (Si${}_{3}$N${}_{4}$); and (**d**) amorphous silicon (A-Si).

A simple hybrid etalon with three deposited layers is presented and studied first. Figure 2a shows the design of ternary hybrid etalons with two asymmetric cavities. Cavity I comprises an A-Si layer with cavity thickness *d* and is sandwiched between Si${}_{3}$N${}_{4}$ and a substrate (SiO${}_{2}$) from both ends, respectively. Cavity II comprises an A-Si layer with cavity thickness *d* and is between Si${}_{3}$N${}_{4}$ and air. Here, the thickness of Si${}_{3}$N${}_{4}$ is given as h.

For simulating the design, an FDTD tool (FDTD Solutions, Lumerical Inc., Vancouver, BC, Canada) [39] was employed. A TM polarized plane wave source was perpendicularly injected from the top of the device, i.e., along the “−Z” direction. The direction of polarization was parallel to the plane of incidence, and the magnetic field was along the y-axis. As per the requirement of the proposed design, the wavelength of the plan wave varied from 250 to 2500 nm (UV-NIR). In order to reduce computational time, the boundary conditions for the x and y axes were antisymmetrical and symmetrical, respectively. However, perfectly matched layers (PML) were used along the z-axis, and the mesh size was 4 nm in all directions.

The fabrication process of thin films is a single-step process and does not require complicated fabrication steps. Figure 2b shows the scanning electron microscopy (SEM) image of the deposited hybrid etalon with d=40 nm and h=100 nm. It illustrates the difference between the simulated and SEM obtained thicknesses of the filter. The refractive indices (n,k) for A-Si and Si${}_{3}$N${}_{4}$ are plotted in Figure 2c,d, respectively.

**Figure 3:**
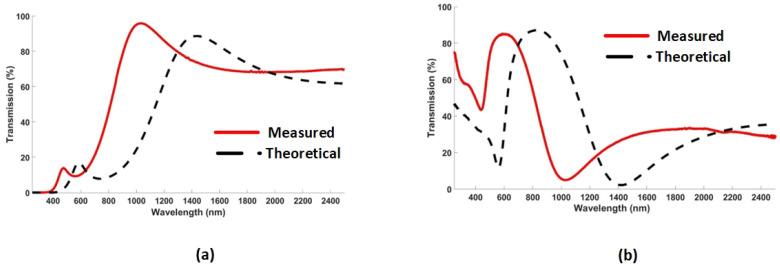
Theoretical (dotted line) and measured (solid line) results for (**a**) transmission and (**b**) reflection spectra of hybrid etalon with *d* = 40 nm and *h* = 100 nm.

Figure 3a,b illustrate the transmission and reflection spectra of the hybrid etalon, respectively. The measured and simulated (also referred as theoretical ) transmission and reflection spectra for a hybrid etalon show the discrepancy in obtained results.

**Figure 4:**
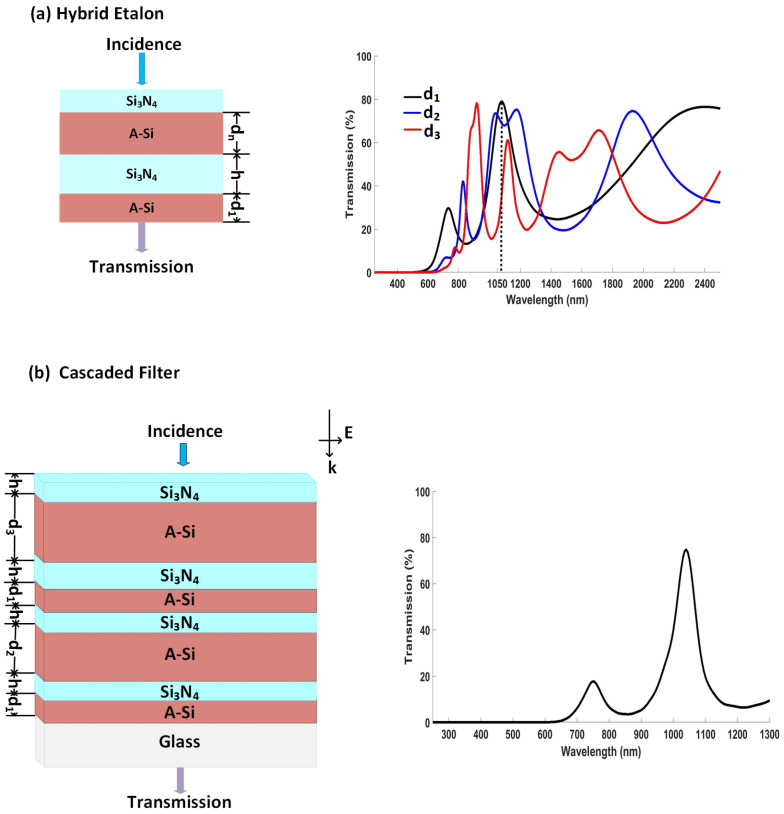
(**a**) Schematic of hybrid etalon and its transmission spectrum at different cavity thicknesses such that $d_{n}=d_{1}$, $d_{2}$, and $d_{3}$, where $d_{1}=d=80$ nm is the fundamental cavity thickness, while $d_{2}$ and $d_{3}$ are its multiples; (**b**) schematic of multilayered thin-film cascaded filter and transmission spectrum of its theoretical and experimental results, where $d_{1}=d=80$ nm is the fundamental thickness of cavity, while $d_{2}$ and $d_{3}$ are its multiples.

The impact of the thickness of one of the cavities on the transmission spectrum of the hybrid etalon was also studied, as shown in Figure 4a. Cavity I comprises an A-Si layer with cavity thickness $d_{1}=80$ nm and was sandwiched between Si${}_{3}$N${}_{4}$ and substrate (SiO${}_{2}$) from both ends, respectively. Cavity II comprises an A-Si layer with cavity thickness $d_{n}$ (multiples of $d_{1}$) and was sandwiched between Si${}_{3}$N${}_{4}$ from both ends. Here, the thickness of Si${}_{3}$N${}_{4}$ h=100 nm. It can be seen that, at $d_{1}=d=80$ nm, there was a significant decrease in free spectral range with an increase in the cavity thickness [26], which resulted in constructive interference at a distinct peak with $\lambda_{res}$ = 1050 nm. However, the sidebands were inevitable.

To suppress the sidebands, two-hybrid etalons were cascaded, as shown in Figure 4b. The obtained results, due to cascaded filter, demonstrated a distinct peak at 1050 nm with 70% efficiency, FWHM of 77 nm, and well-suppressed sidebands. This is accredited to an increase in destructive interference from transmission spectrum of $d_{1}$, $d_{2}$ and $d_{3}$, which occurs everywhere in the VIS-NIR spectrum, other than the desired resonant wavelength $\lambda_{res}$.

**Figure 5:**
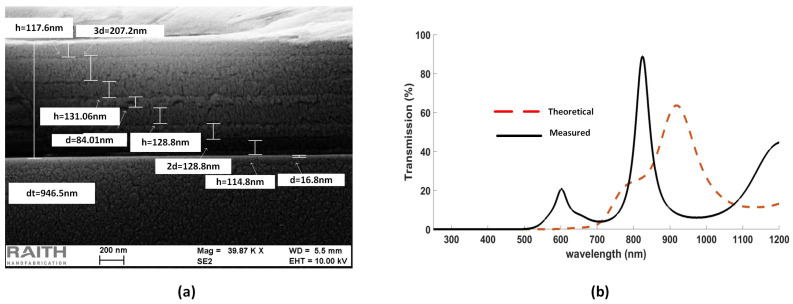
(**a**) SEM image; and (**b**) transmission spectrum of measured and theoretical results of multilayered thin-film cascaded filter.

Figure 5a shows an SEM image of the deposited films and Figure 5b demonstrates the theoretical and measured transmission spectra of multilayered cascaded filter. It is obvious from the obtained graph that the thicknesses observed with the help of SEM not correct, and the theoretical results are not in agreement with the measured results.

## 3. Results and Discussion

The discrepancy in the simulated and experimental results of hybrid etalon (Figure 3) and cascaded filter (Figure 5) is accredited to the difficulty in obtaining the thicknesses of the deposited films through SEM, due to the same average atomic mass of the used materials. Hence, spectroscopic ellipsometry (SE) was employed to obtain the thicknesses of the deposited films.

In this section, the methodology adopted to obtain layer thicknesses of cascaded layers of spectroscopic ellipsometry (SE) is explained. Furthermore, the results are evaluated at different incident and polarization angles of incident light.

The tool available for film characterization was not capable of capturing results at the various incident and polarization angles; therefore, the technique of spectroscopic ellipsometry (SE) [40] was adopted to acquire the desired results.

The essential steps required to derive results through ellipsometry are discussed first here.

**Figure 6:**
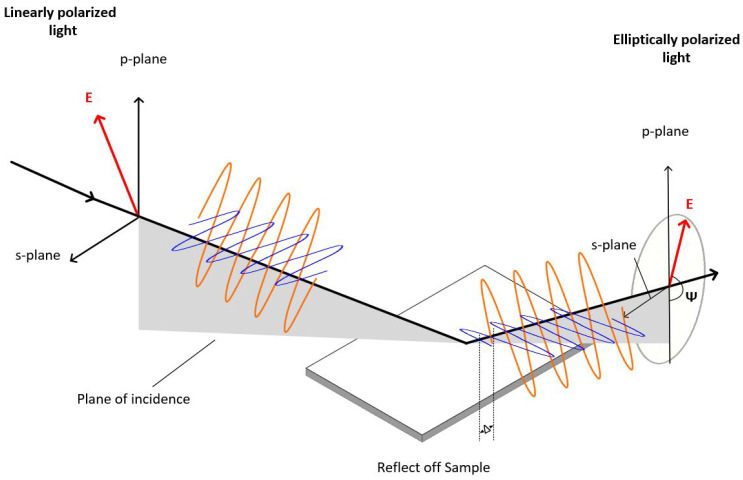
The ellipsometric experiment’s measurement geometry. In the p-s coordinate system, the coordinate system is used to describe the ellipse of polarization. The s direction is defined as parallel to the sample surface and perpendicular to the direction of propagation. The p direction is assumed to be perpendicular to the direction of propagation and lies inside the plane of incidence. The plane of incidence (gray area) is defined as the plane including the input and output beams and the direction which is normal to the sample surface.

As the light interacts with a sample’s structure, its polarization changes (Figure 6). This change in polarization is represented by Psi (ψ) (reflection amplitude ratio angle of incident TM polarization and TE polarization) and Delta (Δ) (phase difference between the TM and TE polarization).

In Figure 6, the plane of incidence (gray area) is defined as the plane including the input and output beams, as well as the direction which is normal to the sample surface. The polarization of incident light is given as electric fields parallel (p or TM mode) and perpendicular (s or TE mode) to the plane of incidence [40].

Mathematically, this is expressed by an amplitude ratio (*tan*ψ) as well as a phase difference (Δ)

(5) $$\tan\left(\psi \right)e^{\iota \Delta }=\frac{{\tilde{r}}_{p}}{{\tilde{r}}_{s}},$$

where ${\tilde{r}}_{p}$ and ${\tilde{r}}_{s}$ are the Fresnel reflection coefficients for p- and s-polarized light, respectively.

**Figure 7:**
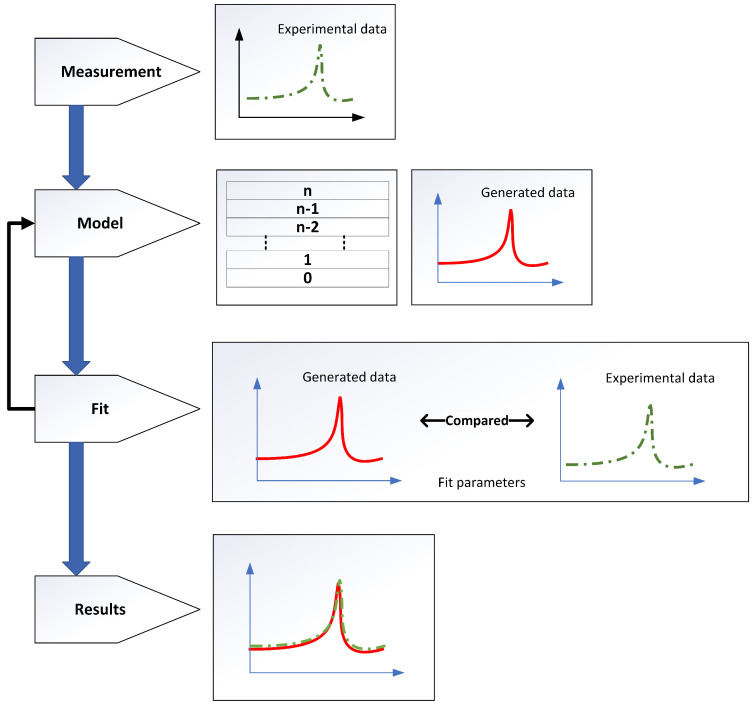
Flow chart for ellipsometry data analysis.

An ellipsometer was used to collect the ellipsometric parameters (ψ, Δ) within a given spectral range and incident angles. However, the measured data (ψ, Δ) do not provide direct information. A data analysis procedure was required to extract meaningful physical information about the sample. The following steps are usually included in the data analysis procedure [41,42], as outlined in the flow chart in Figure 7.

**Figure 8:**
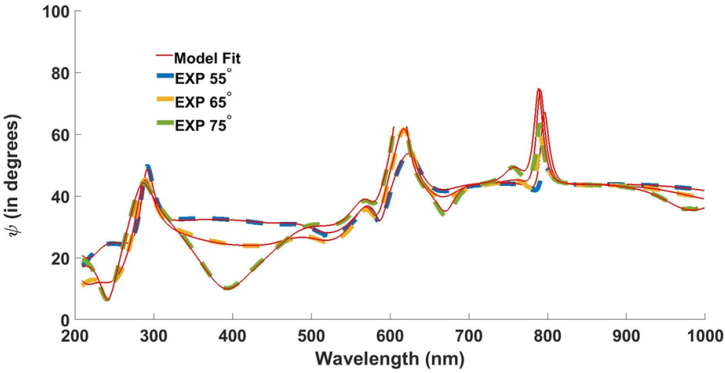
Ellipsometric fit to the proposed design extracted at $55^{{}^{\circ}}$, $65^{{}^{\circ}}$, and $75^{{}^{\circ}}$.

1. The first step is to acquire the experimental data of the sample. In this particular case, a Perkin Elmer Lambda 1050 UV-Vis-NIR spectrophotometer was used to collect the transmission and reflection experimental data.
2. The second step is to create an accurate optical model of the sample system. Here, every material in the system, such as the substrate and the A-Si and Si${}_{3}$N${}_{4}$ layers, was viewed as a “layer”. This model was used to generate SE data.
3. In the third step, the model fit parameters (such as n&k values of each layer, thickness etc.) are defined, and the software adjusts them automatically to improve the agreement between measured and model-generated SE data. This is referred to as “fitting” the data.
4. The fit’s outcomes are assessed. If the results are not satisfactory, the optical model and/or fit parameters are modified, and the data is re-fitted. Mean-squared error (MSE) is a parameter that evaluates the quality of the match between the generated and the experimental data [43]. Mathematically, it is given as
  (6) $$MSE=\sqrt{\frac{1}{2N-M}\sum_{i=1}^{N}\left[{\left(\frac{\psi_{i}^{mod}-\psi_{i}^{exp}}{\sigma_{\psi ,i}^{exp}}\right)}^{2}+{\left(\frac{(\Delta_{i}^{mod}-\Delta_{i}^{exp}}{\sigma_{\psi ,i}^{exp}}\right)}^{2}\right]},$$
  where *N* is the number of (ψ,Δ) pairs, *M* is the number of variable parameters in the model, and σ is the standard deviation on the experimental data points. *MSE* should be positive and near-zero for the maximum likelihood estimate. Figure 8 shows the model fitted to the experimental data with mean-squared error (*MSE*) = 30.51, when the light is incident at $55^{{}^{\circ}}$, $65^{{}^{\circ}}$, and $75^{{}^{\circ}}$ angles.

The obtained thicknesses of the cascaded filter via SE are provided in Table 2.

**Figure 9:**
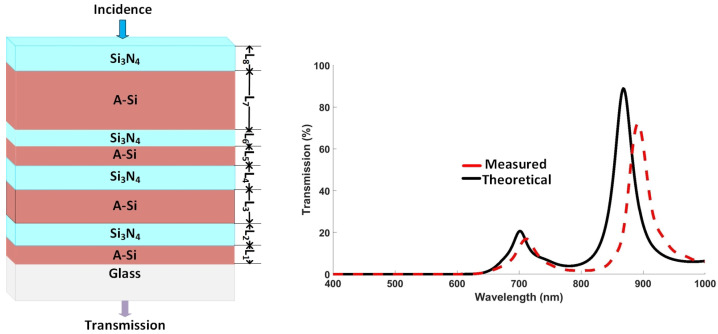
Theoretical and experimental results for multilayered cascaded thin-film filter.

The theoretical plot obtained with the help of layers’ thicknesses in Table 2 are plotted and compared with the experimental results in Figure 9.

**Figure 10:**
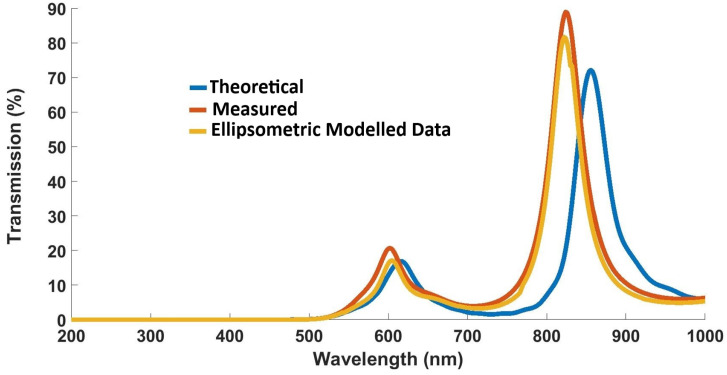
Transmission spectrum produced with the help of simulated, experimental, and ellipsometry-derived data.

Figure 10 presents the transmission spectrum of the multilayered cascaded thin film obtained with the help of simulations/theoretical, measured, and spectroscopic ellipsometric data. It is evident from the produced results that the thicknesses (Table 2) produced via SE are close to the thicknesses of the deposited films.

The designed devices were simulated at different incident angles, i.e., from $0^{{}^{\circ}}$ to $70^{{}^{\circ}}$ in steps of $10^{{}^{\circ}}$. Moreover, the results at each angle were compared and analyzed.

**Figure 11:**
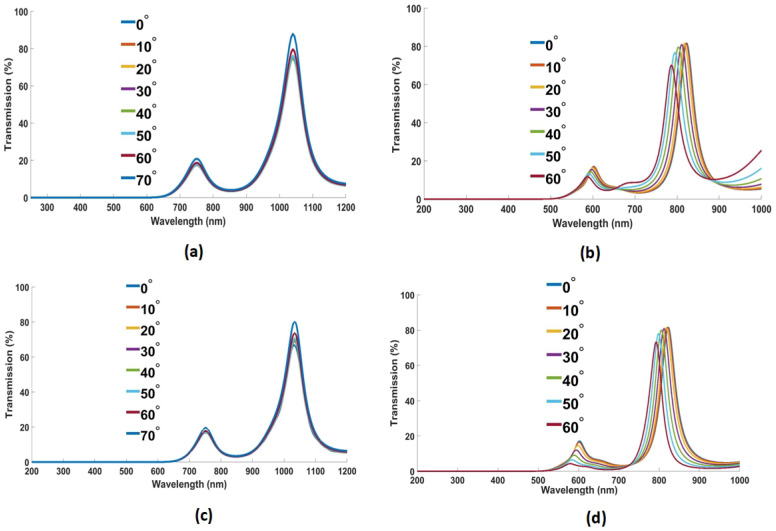
Simulated results of the designed filter at different source incident angles in (**a**) TM and (**c**) TE polarization mode. Transmission profile at different incident angles the incident light in (**b**) TM and (**d**) TE mode obtained with the help of spectroscopic ellipsometry.

Figure 11a,c show the impact of incident angle (θ) of incident light on the device’s performance, where light polarization is in TM and TE mode, respectively. It can be deduced from the obtained simulated plots in Figure 11a,c that the transmission spectrum remains unchanged till $60^{{}^{\circ}}$. However, the results obtained through spectroscopic ellipsometry, in Figure 11b,d, show angle invariance up to $50^{{}^{\circ}}$.

**Figure 12:**
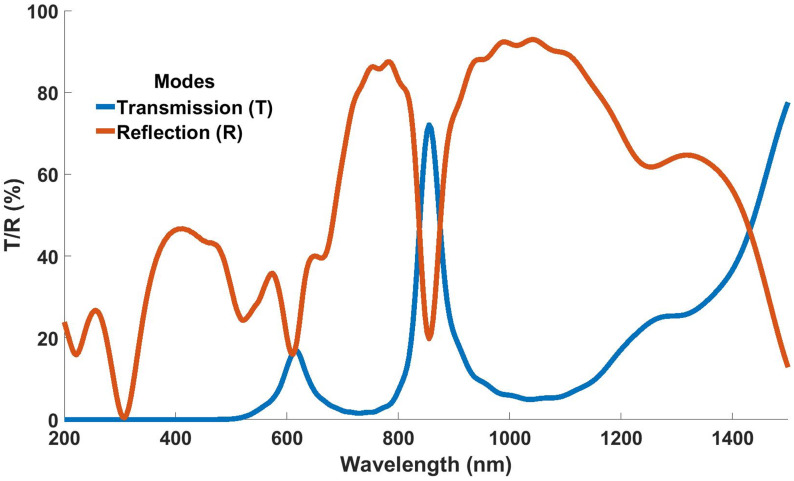
Response of cascaded multilayered thin-film filter in transmission and reflection mode.

The design was further evaluated for its operation in reflection mode. The results in Figure 12 show that the cascaded multilayered thin-film filter is capable of operating in transmission and reflection modes.

### 3.1. Fabrication

PECVD technology was used to deposit a multilayered thin-film cascaded filter. A 5 mm-thick adhesive layer of SiO${}_{2}$ was used during a deposition for better adhesion between the A-Si and Bk7 glass substrates. The design was fabricated at Australian National Fabrication Facility (ANFF) UNSW, Sydney, Australia. The depositions were carried out using an Oxford instrument system 100 PECVD module at 180 °C on a BK7 substrate, without opening the chamber.

### 3.2. Film Characterization

The transmission and reflection spectrum of the design were obtained with the help of the Perkin Elmer Lambda 1050 UV-Vis-NIR Spectrophotometer. Due to the tool’s constraints, the angle invariance of the design in TM and TE mode was determined by employing SE. The data acquisition software for extracting results through SE was J.A. Woollam variable-angle spectroscopic ellipsometry (WVASE), manufactured by J.A Woollam Co., Inc., Lincoln, NE 68508, USA.

## 4. Conclusions

In conclusion, an all dielectric transreflective angle-insensitive NIR filter was demonstrated. The filter comprises only eight layers of A-Si and Si${}_{3}$N${}_{4}$, successively deposited on a glass substrate. The simulated results produced transmission up to 70% and FWHM of 77 nm, with the insensitivity of incident angle up to $60^{{}^{\circ}}$. The experimental results are in unison with the simulated results. However, the thicknesses obtained by scanning electron microscopy are in disagreement with the deposited thicknesses, due to usage of materials with approximately same average atomic mass, i.e., A-Si, Si${}_{3}$N${}_{4}$, and SiO${}_{2}$.

Overall, the simple design, high efficiency, and CMOS compatibility of the proposed filter make it a suitable candidate for image sensing devices. By adding another hybrid etalon to the current design, we can obtain a sharper resonance with low intensity, that can be useful for label-free biosensing, where high-refractive-index sensitivity is desirable.

For future work, we plan to further manipulate the number of layers and/or material of layers to achieve resonance at other wavelengths of interest across the NIR region.

## Figures and Tables

**Table 1:** Summary of literature review.

Ref.	Design	Fabrication Technique
[22]	GMR-based 2D A-Si nanodisk array embedded in SiO${}_{2}$.	EBL and PECVD.
[31]	Ti nanodisk-shaped array with Ti/Au/SiO${}_{2}$.	E-beam evaporation and RF sputtering.
[32]	MgF${}_{2}$ nanodisk-shaped array with MgF${}_{2}$/Au/SiO${}_{2}$.	E-beam evaporation and EBL.
[33]	A single aperture surrounded by concentric periodic corrugations on a silver (Ag) thin film for simultaneous imaging of a spectral range from the visible to the near-infrared	EBL, vacuum evaporation, and an FIB.
[29]	Triangular lattice of Al nanodisks with Al/SiO${}_{2}$.	NIL and subsequent lift-off process.
[18]	Symmetry-breaking elliptical nanoholed Au sheet on SiO${}_{2}$, with FWHM of 79 nm and efficiency up to 44%.	EBL and annealing.
[34]	Au circular nanohole sheet on a GaAs substrate for infrared filtering.	EBL and e-beam evaporation.
[17]	GMR-based 1D Si${}_{3}$N${}_{4}$ grating on soda lime substrate for infrared filtration.	Contact photolithography and PECVD.
[26]	Hybrid cascaded multilayered thin-film filter Si/SiO, for mid-infra-red filtration.	E-beam and resistance-heating methods.
[28]	Cascaded thin-film filter (A-Si/SiO${}_{2}$), provided sharp roll-off, and 130 nm bandwidth.	PECVD
[27]	Cascaded thin-film filter (A-Si/SiO${}_{2}$), investigated to filter the red color (620–750 nm).	PECVD
[30]	Multilayered thin-film filter (A-Si/Si${}_{3}$N${}_{4}$) studied for blocking the visible region (400–700 nm).	PECVD
[This work]	Hybrid, cascaded, thin-film filter (A-Si/Si${}_{3}$N${}_{4}$) for NIR filtering with an FWHM of 77 nm.	PECVD

PECVD—plasma enhanced chemical vapor deposition; EBL—electron beam lithography; NIL—nanoimprint lithography; FIB—focused ion beam.

**Table 2:** Thicknesses of deposited films extracted with the help of ellipsometry.

Layer Numbers	Symbol	Material	Thicknesses (nm)
1	L${}_{1}$	A-Si	54.730
2	L${}_{2}$	Si${}_{3}$N${}_{4}$	84.430
3	L${}_{3}$	A-Si	112.557
4	L${}_{4}$	Si${}_{3}$N${}_{4}$	112.043
5	L${}_{5}$	A-Si	52.043
6	L${}_{6}$	Si${}_{3}$N${}_{4}$	106.518
7	L${}_{7}$	A-Si	165.611
8	L${}_{8}$	Si${}_{3}$N${}_{4}$	101.408

## Data Availability

Not applicable.

## Abbreviations

The following abbreviations are used in this manuscript:

ANFF	Australian National Fabrication Facility
CMOS	complementary metal-oxide semiconductor
EBL	electron beam lithography
FOM	figure of merit
GMR	guided mode resonance
MSE	mean-squared error
NIR	near infrared
PECVD	plasma-enhanced chemical vapor deposition
PML	perfectly matched layers
SE	spectroscopic ellipsometry
SEM	scanning electron microscopy
TE	transverse electric
TM	transverse magnetic
WVASE	Woollam variable-angle spectroscopic ellipsometry
